# Impact of point-of-care CD4 testing on linkage to HIV care: a systematic review

**DOI:** 10.7448/IAS.17.1.18809

**Published:** 2014-01-20

**Authors:** Elke Wynberg, Graham Cooke, Amir Shroufi, Steven D Reid, Nathan Ford

**Affiliations:** 1 Department of Infectious Diseases, Faculty of Medicine, Imperial College London, South Kensington, London, UK; 2 Médecins Sans Frontières, Cape Town, South Africa; 3 HIV/AIDS Department, World Health Organization, Geneva, Switzerland

**Keywords:** antiretroviral therapy, HIV/AIDS, point-of-care CD4, retention, treatment initiation

## Abstract

**Introduction:**

Point-of-care testing for CD4 cell count is considered a promising way of reducing the time to eligibility assessment for antiretroviral therapy (ART) and of increasing retention in care prior to treatment initiation. In this review, we assess the available evidence on the patient and programme impact of point-of-care CD4 testing.

**Methods:**

We searched nine databases and two conference sites (up until 26 October 2013) for studies reporting patient and programme outcomes following the introduction of point-of-care CD4 testing. Where appropriate, results were pooled using random-effects methods.

**Results:**

Fifteen studies, mainly from sub-Saharan Africa, were included for review, providing evidence for adults, adolescents, children and pregnant women. Compared to conventional laboratory-based testing, point-of-care CD4 testing increased the likelihood of having CD4 measured [odds ratio (OR) 4.1, 95% CI 3.5–4.9, n=2] and receiving a CD4 result (OR 2.8, 95% CI 1.5–5.6, n=6). Time to being tested was significantly reduced, by a median of nine days; time from CD4 testing to receiving the result was reduced by as much as 17 days. Evidence for increased treatment initiation was mixed.

**Discussion:**

The results of this review suggest that point-of-care CD4 testing can increase retention in care prior to starting treatment and can also reduce time to eligibility assessment, which may result in more eligible patients being initiated on ART.

## Introduction

There is a recognized need for improving the care pathway from HIV diagnosis to timely antiretroviral therapy (ART) initiation, with several recent studies highlighting substantial losses in the continuum of care from HIV testing to ART initiation [1–3]. Reasons reported for such attrition vary and include long waiting times at clinics, concerns about drug side effects, lack of CD4 testing and delays in receiving CD4 results [4].

Losses to care during the pre-ART period are of greatest concern for those individuals in clinical need of ART; timely identification of ART-eligible individuals is a critical step in the care pathway. According to a recent review from sub-Saharan Africa, approximately one-quarter of patients are lost to care in the step between testing HIV-positive and having a CD4 measurement done [1].

One proposed approach to improving access to CD4 testing and reducing delays in eligibility assessment is the use of point-of-care (PoC) CD4 technologies. Traditional laboratory-based methods for CD4 measurement rely on an infrastructure for transporting blood samples to a centralized facility that is often far removed from remote testing centres. In addition, flow cytometry requires technical expertise, complex instrumentation and software, and a reliable data management system to ensure the results are returned promptly to the health worker and patient. A number of PoC CD4 machines are currently available on the market, with more expected in the coming years [5]. Early results show that PoC CD4 can reduce time to eligibility assessment and losses to care prior to ART initiation, and the approach is promoted by the World Health Organization (WHO) as a way to improve access at peripheral sites [6].

We undertook this systematic review to evaluate the programme impact of PoC CD4 testing, particularly with respect to retention in pre-ART care and to time to assessment for ART eligibility.

## Methods

This systematic review was conducted according to a study protocol (Supplementary file) following the requirements of the Preferred Reporting Items for Systematic Reviews and Meta-Analyses (PRISMA) Statement [7].

### Search strategy

We developed a compound search strategy that combined terms for *HIV*, *point-of-care*, *CD4* and *test* (including MeSH terms) and the names of specific PoC CD4 technologies listed in the UNITAID HIV/AIDS Diagnostic Technology Landscape Report [5]. Several different definitions of PoC testing have been proposed [8]. In addition to PoC, terms such as *portable*, *remote* and *mobile* were included in the initial search. For this review, we applied a working definition of PoC CD4 testing as the rapid enumeration of CD4 levels with a technology suitable for remote settings that allows results to be available to the patient on the same day as testing. All included studies had to specify that PoC technology was used.

We searched for studies published between 1 January 2005 (the beginning of the period when PoC CD4 technologies were first being trialled) and 15 March 2013, in nine electronic databases (EMBASE, Ovid MEDLINE, LILACS, WHOLIS, ADOLEC, MedCarib, IBACS, CidSaude and PAHO). This search was updated in PubMed to 26 October 2013. Titles were screened by one reviewer (EW) and the final selection of studies was carried out in duplicate (EW, NF). We also searched the electronic databases of the Conference on Retroviruses and Opportunistic Infections (up to Atlanta, GA, March 2013) and International AIDS Society Conferences (up to Kuala Lumpur, June 2013), reviewed bibliographies of review articles and contacted experts in the field. Finally, manufacturers of PoC CD4 tests were contacted for information about ongoing trials. We included randomized and non-randomized comparative trials, observational comparative and non-comparative studies, qualitative studies that reported patient and provider satisfaction, and cost-effectiveness studies. No age, language or geographical restrictions were applied.

### Data extraction and synthesis

The primary outcomes of interest were the proportion of patients retained in care at each step of the HIV-care pathway and for those remaining in care, the time it took patients to reach the next step in the care pathway. The following steps were evaluated: (1) HIV diagnosis (i.e. confirmation of status) to CD4 testing; (2) CD4 testing to delivery of CD4 results (i.e. eligibility assessment); and (3) eligibility assessment to ART initiation for those eligible. Where possible, results were compared against standard of care: laboratory-based CD4 level counting, clinical staging of patients using WHO criteria or both.

Data extraction was conducted by one reviewer (EW) and verified by a second (NF) using pre-constructed extraction forms. We further assessed the quality of included studies using a pre-defined quality assessment framework (see study protocol in Supplementary file).

The proportion of patients progressing from one step of the care pathway to the next was estimated together with corresponding 95% confidence intervals (CIs). Depending on whether patients received PoC CD4 or standard of care, odds ratios (ORs) and corresponding 95% CIs were calculated to compare the likelihood of achieving each step in the care cascade. These outcomes were then pooled using a DerSimonian–Laird random-effects model [9]. Between-study heterogeneity was estimated using the τ^2^ statistic [10]. All data were analyzed using STATA version 12.0.

## Results

### Study characteristics

**Figure 1:**
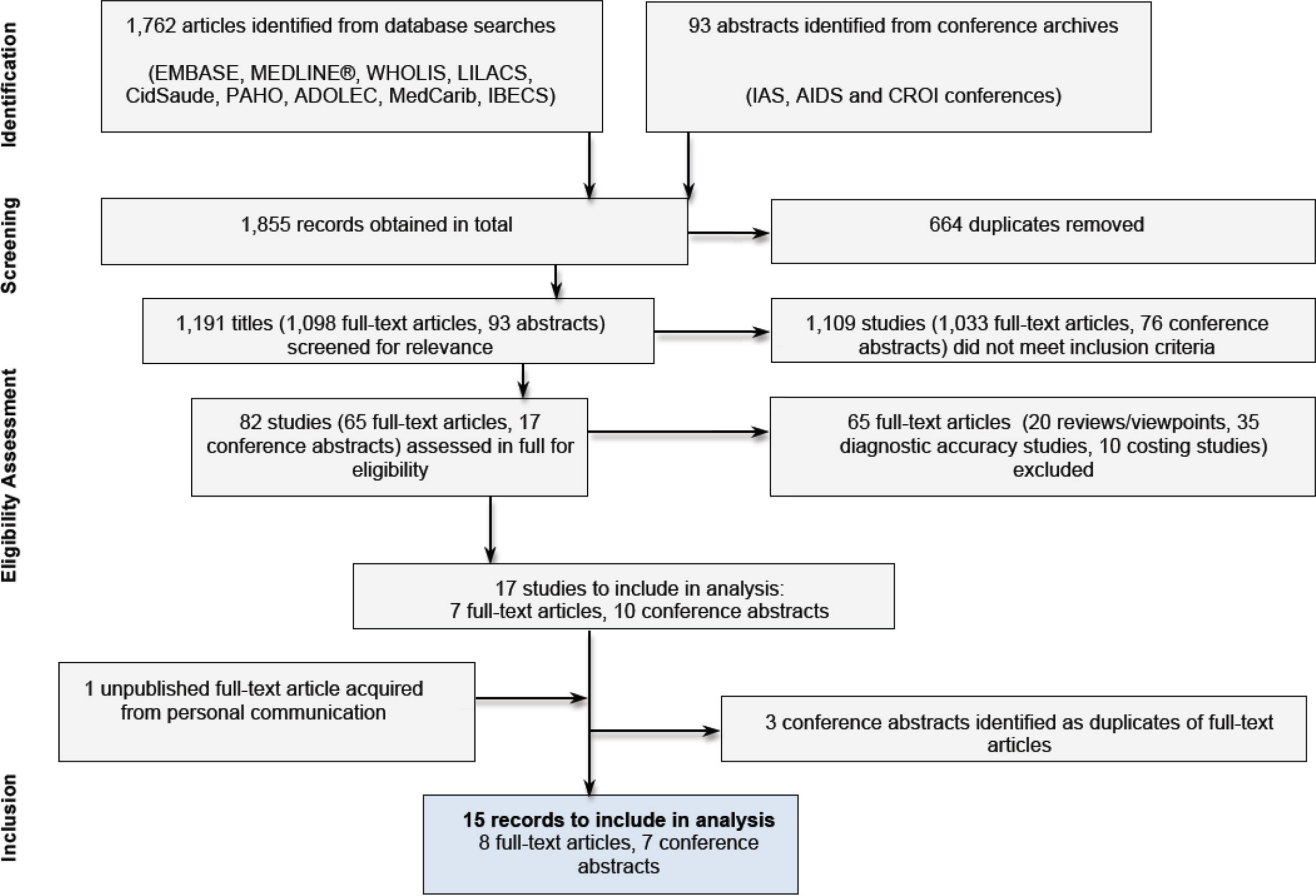
Study selection process

From an initial 1840 titles, 15 eligible studies were identified, comprising eight full-text articles [11–14] and seven conference abstracts (Figure 1). The conference abstracts were checked for publication as full-text articles at the time that the final search was conducted (26 October 2013). The majority of studies were carried out in southern Africa, including eight in South Africa, two in Mozambique and two in Zimbabwe. All studies included adults in their cohort, two studies also included children, three included adolescents, three focussed on pregnant women and one looked specifically at migrants. All studies were published either as full texts or as conference abstracts between 2011 and 2013. Studies varied in type of clinical setting (permanent or mobile), geographical location (rural, urban, or peri-urban) and patient care status (newly diagnosed or in long-term follow-up) (Table 1).

**Table 1:** Characteristics of included studies

	First author	Year	Publication	Study type	Country	Setting	Clinic type	Study population	Size	ART threshold	Device
1	MSF	2013	Unpublished	Observational cohort (before-after)	South Africa	Rural	Mobile HCT	HIV+, ART-eligible, age >1	354	<250 up to end August 2011, <350 thereafter	PIMA
2	Larson	2013	Full text	Cross-sectional	South Africa	Urban	HIV clinic	All HIV+	897	<200 up to end August 2012, <350 thereafter	BD FASCount
3	van Rooyen	2013	Full text	Observational cohort	South Africa	Rural	Mobile HBCT	HIV+, age>18	201	<200, <350 for pregnant women	PIMA
4	Patten	2013	Full text	Observational cohort (before-after)	South Africa	Peri-Urban	Youth HIV clinic	HIV+, age 14–25	576	<250 up to end August 2011, <350 thereafter	PIMA
5	Black	2013	Full text	Observational cohort	South Africa	Urban	Community antenatal Clinic	Pregnant women	3479	<350 or WHO stage III/IV	PIMA
6	Rioja	2013	Conference abstract	Observational cohort (before-after)	Cameroon	Rural	District hospital	All HIV+	1513	Not stated	PIMA
7	Brouillette	2013	Conference abstract	Retrospective cohort	Uganda	Diverse	Diverse	All HIV+	Not stated	Not stated	PIMA
8	Mwanja	2013	Conference abstract	Observational cohort (before-after)	Tanzania	Diverse	Primary healthcare facility	All HIV+	Not stated	<350	PIMA
9	Larson	2012	Full text	Non-randomized trial	South Africa	Rural	Mobile HBCT	HIV+, age>18	508	Not stated	PIMA
10	De Schacht	2012	Conference abstract	Observational cohort (before-after)	Mozambique	Diverse	HIV clinic	HIV+ pregnant women	3410	Not stated	n.s.
11	Matambo	2012	Conference abstract	Observational cohort (before-after)	South Africa	Rural	Mobile HIV, TB and PHC	HIV+ migrant farm workers	2906	<200 up to end 2011, <350 thereafter	PIMA
12	Muchedzi	2012	Conference abstract	Observational cohort (before-after)	Zimbabwe	Diverse	PMTCT Site	HIV+ pregnant women	2310	Not stated	n.s.
13	Jani	2011	Full text	Observational cohort (before-after)	Mozambique	Rural & Peri-Urban	Primary healthcare facility	HIV+, age>1	534	<250 age>15; <350 age 4–14; <750 age 1–3	PIMA
14	Faal	2011	Full text	Randomized controlled trial	South Africa	Urban	Primary healthcare facility	HIV+, age>18	344	<215	BD FASCount
15	Hatzold	2011	Conference abstract	Observational cohort (before-after)	Zimbabwe	Diverse	HIV clinic	HIV+, ART-eligible	182	Not stated	n.s.

### Study quality assessment

Overall, the quality of included studies was considered to be low to moderate (Supplementary file). Only one of the two comparative trials was randomized, and neither reported staff training. Among the observational cohort studies, six studies were retrospective, five were prospective and two were mixed. Only two studies reported a sample size calculation. Studies did not always explicitly report whether capillary or venous blood samples were used. One study was supported by the test manufacturer (Alere) but stated that the donor played no role in the conduct of the study. Assessment of study quality was limited by the large number of conference abstracts that did not report methodological criteria in detail.

### Impact on the care pathway

**Figure 2:**
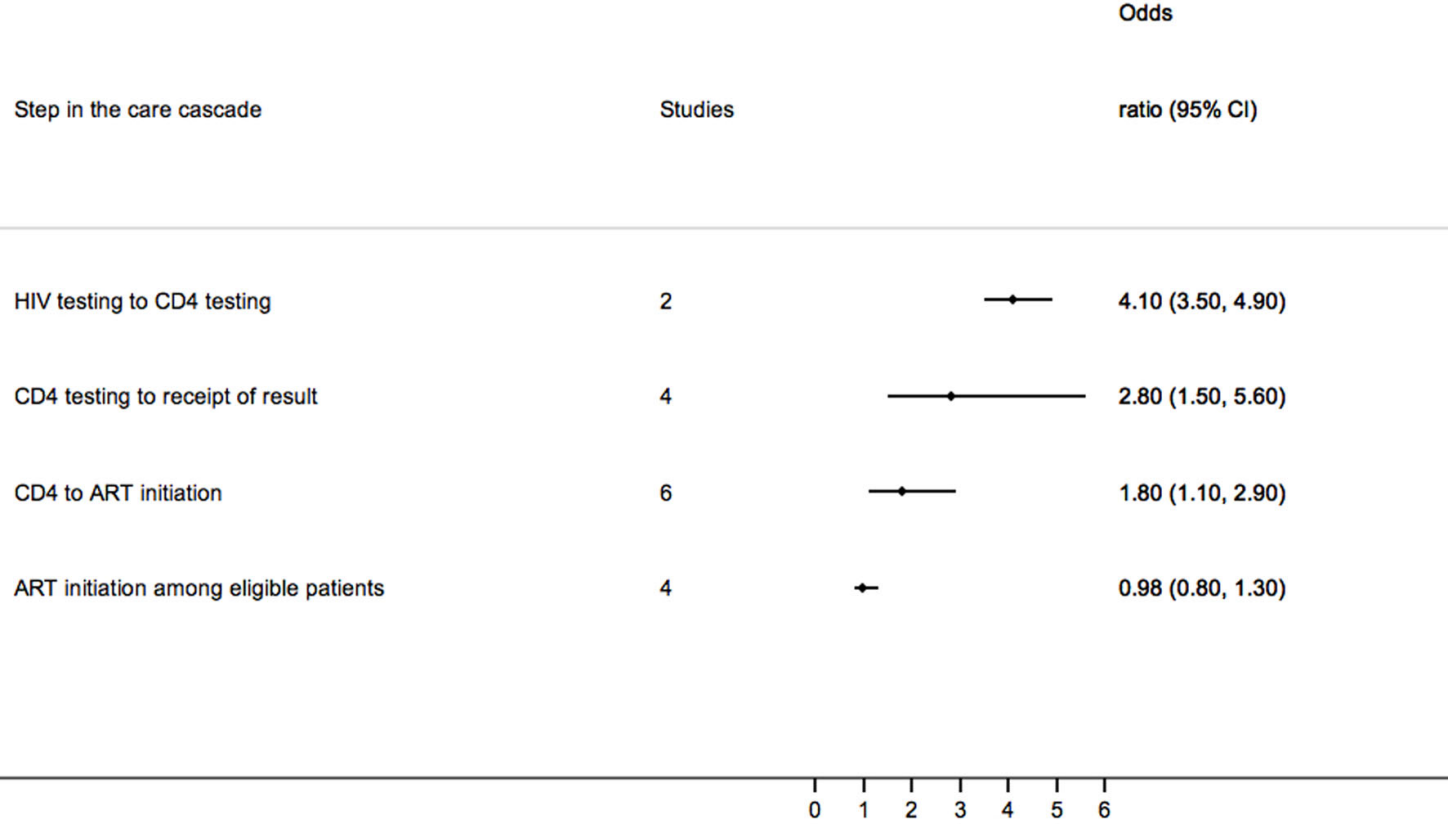
Pooled odds ratio of achieving the next step in the treatment cascade comparing point of care CD4 testing against standard of care

Two studies provided data on the impact of PoC CD4 from HIV testing to eligibility assessment, and overall the likelihood of being tested for CD4 increased fourfold (OR 4.1, 95% CI 3.5–4.9) [15, 16]. The likelihood of people receiving their result after being tested for CD4 was also increased (four studies: OR 2.8, 95% CI 1.5–5.6) (Figure 2).

Among those assessed as being eligible for ART, six studies reported the proportion of people who were initiated on treatment [11, 12, 16–19]. In a randomized trial from South Africa, people receiving PoC CD4 testing were almost three times more likely to be initiated on ART compared to standard of care (OR 2.8, 95% CI 1.4–5.7) [11]. A second study, of migrant farm labourers in South Africa, reported that ART-eligible patients were more than six times likely to initiate ART in the period following the introduction of PoC CD4 testing compared to the period before PoC testing was available (OR 6.3, 95% CI 3.9–10.3). In this study, time to ART initiation following clinic registration was 14 days in the post PoC period [interquartile range (IQR) 14–31 days] compared to 143.5 days in the pre-PoC period (IQR 35–287 days) [18]. In a third study, from South Africa, there was no difference in the proportion of eligible patients initiating ART within 16 weeks (1.07, 95% CI 0.89–1.29) [19]. A fourth study, from Cameroon, found that the number of patients initiated on ART was significantly higher after the introduction of PoC CD4 (from 33% to 87%) despite a similar proportion of patients being eligible for ART (49% vs. 46%) [20].

Overall, the likelihood of initiating ART was greater when PoC CD4 was used (OR 1.8, 95% CI 1.1–2.9); however, when this analysis was limited to those studies that reported this outcome for eligible individuals, there was no difference between PoC and standard of care (OR 1.0, 95% CI 0.8–1.3).

Completion of all the steps between HIV diagnosis and ART initiation was assessed by two studies. Both studies reported a reduction in time to ART initiation with the PoC CD4 testing group. This was statistically significant in one study [12] where median (IQR) days from enrolment to ART initiation was reduced from 48 (34–80) to 20 (10–31) days. In the second study [17] median (IQR) days from enrolment to ART initiation was reduced from 35 (22–57) to 26 (19.5–33.5) days.

## Discussion

In this review, PoC CD4 was found to be associated with an increased likelihood that patients would achieve CD4 testing and eligibility assessment compared to laboratory-based testing, and it was found to reduce the time intervals at these critical steps in the care pathway. There was also evidence that use of PoC testing could increase the likelihood of HIV-positive individuals being initiated on ART in some settings.

The studies included in this review were conducted within a range of settings and patient populations. Of note, three of the included studies were done in mobile clinics, suggesting a role for PoC CD4 testing in extending the scope of services, for example, through linkage with community and household testing campaigns. Several studies have also found that PoC CD4 testing may also be more cost-effective than laboratory-based methods [13, 21–23] and there is also evidence of acceptability among health workers [24]. We did not formally assess cost or performance of PoC CD4 testing, and any benefits of increased linkage to care must be weighed against financial viability and evidence-based accuracy and specificity findings.

An important feature for any diagnostic technology used in rural high HIV-burden settings is simplicity, [25] particularly because ART delivery is increasingly being managed by lower level health workers in decentralized sites [26]. One study [11] reported that the device was successfully used by nurses and other non-physician clinicians, and five studies [12, 15, 18, 27, 28] reported data from rural locations.

Nonetheless, there are several important caveats that should be considered in the interpretation of these findings. Most importantly, the magnitude of improved retention and time to eligibility assessment will be influenced by contextual factors such as distance between place of residence and clinic, and the turnaround time for laboratory results, to which PoC testing was compared. Another limitation is that only one of the studies was fully randomized, and observational studies carry a higher risk of introducing bias (for example, the differential application of technology to different patient groups). Several studies included in this review compared outcomes before and after the introduction of PoC CD4, and the observed changes may have been due to other programme improvements during the reporting period. Diagnostic research tends to focus on test performance rather than patient related outcomes [29] although efforts are underway to carry out high quality studies to address these questions in other disease areas [30] and similar studies would be helpful for PoC diagnostics. Inconsistency in outcome reporting meant that each outcome was assessed by only a subset of studies. Moreover, although the inclusion of conference abstracts is a strength in that it serves to limit publication bias, the limited information reported by abstracts decreases confidence in the quality of these studies and the extent to which they could contribute data to the analyses included in this review. Finally, it should be noted that this review did not assess the performance of different PoC technologies. Current PoC technologies are variously associated with high cost and with discrepant results that are associated with sampling challenges [31]. This review's findings should be considered as indicative of the potential programme benefits of PoC CD4 testing and not as an endorsement of any particular test.

In view of the WHO's new recommendations for treatment eligibility and the use of viral load to monitor treatment failure [32], the benefits of PoC CD4 may change over time. Nevertheless, CD4 cell count determination will remain important for ART eligibility assessment and for baseline disease status.

## Conclusions

The potential for PoC testing to increase health service effectiveness and efficiency is increasingly recognized, particularly for low- and middle-income countries. PoC tests now exist for several infectious diseases, including malaria, syphilis and tuberculosis, with the hope that testing will become more decentralized and less laboratory dependent, while improving accuracy over syndromic management protocols that are often used in such settings [33]. However, the benefit of PoC tests will only be realized if technological innovation is paired with effective implementation [34]. Evaluation of the patient benefit, programmatic impact and cost-effectiveness of PoC technologies forms as important a part of the evidence base as do diagnostic accuracy studies.

In summary, this review lends further support for the use of PoC CD4 in improving time to eligibility assessment, which will contribute to increasing the numbers of eligible patients who receive ART. PoC testing may have particular value for certain population groups who traditionally have faced difficulties in accessing ART such as adolescents, migrant workers and rural populations. Future research should focus on the potential added value of PoC CD4 testing in the monitoring of HIV-positive patients not yet eligible for treatment as well as on the long-term impact of PoC CD4 implementation on patient outcomes. Finally, this review highlights the fact that even with PoC CD4 testing, retention in care is suboptimal [34]. Eligibility assessment must be accompanied by other interventions to ensure successful linkage to an effective treatment programme.
